# Ultrafast Transverse Modulation of Free Electrons by Interaction with Shaped Optical Fields

**DOI:** 10.1021/acsphotonics.2c00850

**Published:** 2022-09-27

**Authors:** Ivan Madan, Veronica Leccese, Adam Mazur, Francesco Barantani, Thomas LaGrange, Alexey Sapozhnik, Phoebe M. Tengdin, Simone Gargiulo, Enzo Rotunno, Jean-Christophe Olaya, Ido Kaminer, Vincenzo Grillo, F. Javier García de Abajo, Fabrizio Carbone, Giovanni Maria Vanacore

**Affiliations:** † Institute of Physics, École Polytechnique Fédérale de Lausanne, Lausanne, 1015, Switzerland; ‡ HOLOEYE Photonics AG, Volmerstrasse 1, 12489 Berlin, Germany; § Department of Quantum Matter Physics, University of Geneva, 1211 Geneva, Switzerland; ∥ Centro S3, Istituto di Nanoscienze-CNR, 41125 Modena, Italy; ⊥ Department of Electrical and Computer Engineering, Technion, Haifa 32000, Israel; # ICFO-Institut de Ciencies Fotoniques, The Barcelona Institute of Science and Technology, 08860 Castelldefels (Barcelona), Spain; ○ ICREA-Institució Catalana de Recerca i Estudis Avançats, Passeig Lluís Companys 23, 08010 Barcelona, Spain; □ Department of Materials Science, University of Milano-Bicocca, Via Cozzi 55, 20126 Milano, Italy

**Keywords:** electron-beam shaping, electron−photon interaction, PINEM, ultrafast transmission electron microscopy, spatial light modulator

## Abstract

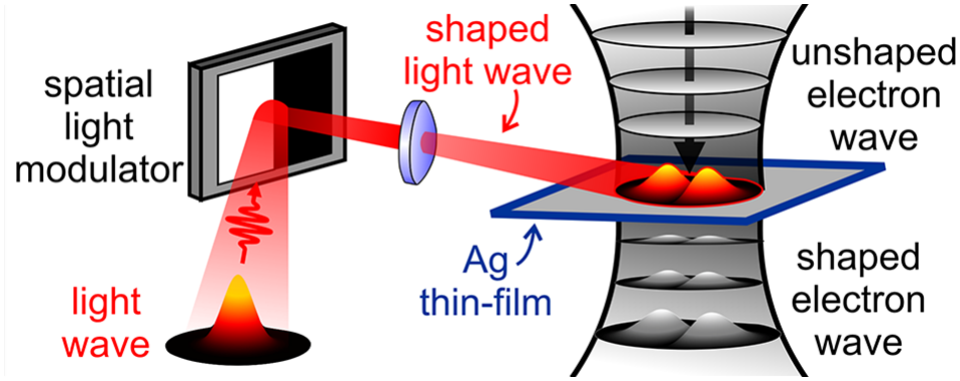

Spatiotemporal electron-beam shaping is a bold frontier of electron microscopy. Over the past decade, shaping methods evolved from static phase plates to low-speed electrostatic and magnetostatic displays. Recently, a swift change of paradigm utilizing light to control free electrons has emerged. Here, we experimentally demonstrate arbitrary transverse modulation of electron beams without complicated electron-optics elements or material nanostructures, but rather using shaped light beams. On-demand spatial modulation of electron wavepackets is obtained via inelastic interaction with transversely shaped ultrafast light fields controlled by an external spatial light modulator. We illustrate this method for the cases of Hermite-Gaussian and Laguerre-Gaussian modulation and discuss their use in enhancing microscope sensitivity. Our approach dramatically widens the range of patterns that can be imprinted on the electron profile and greatly facilitates tailored electron-beam shaping.

For a long time, electron microscopy has epitomized the art of acquiring images at increasingly higher spatial resolution.^1^ Instrumentation research was mainly aimed at obtaining atomic-size probes and aberration-free images. About 10 years ago a different trend was started when the first ideas of electron beam manipulation were introduced.^2−5^ These methods mainly relied on the use of phase and amplitude holograms,^6−12^ as well as electrostatic and magnetostatic phase elements,^13−17^ to coherently modulate the amplitude and phase of the transmitted free-electron wave function.^18−21^ Thanks to such advances, beam shaping can now provide new routes toward image-resolution enhancement, selective probing, low-dose imaging, and depth information, as well as faster data acquisition.^8,22,23^ We anticipate that such advancements will be able to open new frontiers not only in microscopy but also in optoelectronics, quantum information, and biosensing.

To reach the required high speed, flexibility and precise phase control needed to transform this strong potential into a reality, a radical departure from current passive or slowly varying schemes becomes necessary. A swift change of paradigm has recently occurred with the exploration of new approaches based on light-mediated coherent modulation of the longitudinal^24−30^ and transverse^24,31−35^ amplitude and phase of an electron wave function.^36−40^ Such schemes exploit the effect of a strong interaction between free electrons and electromagnetic fields taking place either in free-space through elastic coupling mediated by the ponderomotive force of a standing wave of light^41−43^ (only phase modulation) or in the presence of fabricated nanostructures via inelastic exchange of photon quanta^33,44−50^ (both phase and amplitude modulations when using energy filtering). The use of properly synthesized ultrafast light fields can provide coherent shaping of the electron wave with temporal modulation speed down to the femtosecond range and below, many orders of magnitude faster than in conventional electrically controlled schemes.

Here, we present the first step toward the development of such a photonic free-electron modulator (PELM) and experimentally demonstrate the transverse modulation of electron pulses with a computer-controlled arbitrarily shaped transverse profile of the ultrafast light field. In our approach, which is schematically shown in Figure 1a, instead of using fabricated nanostructures to create a specific light field configuration, we adopt an external spatial light modulator (SLM) to imprint the desired amplitude and phase pattern on the optical field, which is in turn projected on a flat electron-transparent plate. Such a pattern is then imprinted on the electron wavepacket when it crosses the light field via inelastic electron-light scattering. The resulting electron pattern is then observed by energy-filtered ultrafast electron microscopy.^51,52^ Using a SLM thus enormously widens the range of light patterns that can be adopted for electron beam shaping with respect to the limited set of configurations defined by specific fabricated nanostructures. By precisely tuning the phase and intensity of the light field via the SLM, such a method allows us to externally and arbitrarily manipulate the transverse and longitudinal electron distribution with an unprecedentedly high control of electron beams *via* programmable time/energy and space/momentum distributions.

**Figure 1:**
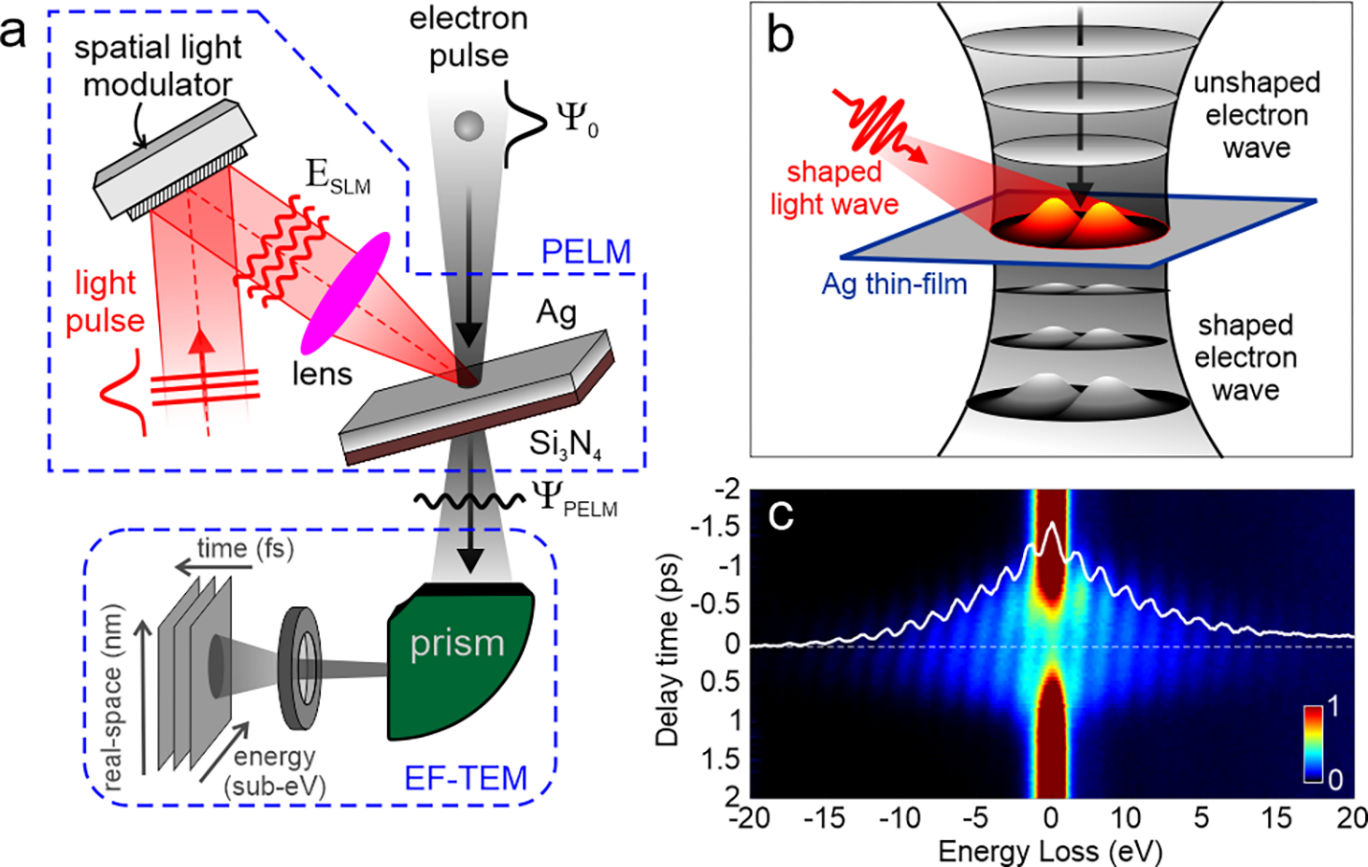
Schematics of a photonic free-electron modulator (PELM). (a) An external spatial light modulator (SLM) is used to imprint an arbitrarily shaped amplitude and phase pattern on the optical field. The light beam is then focused on a thin Ag/Si_3_N_4_ film inside an ultrafast transmission electron microscope (UTEM). The SLM is placed in the conjugate plane with respect to the thin plate. Femtosecond electron pulses in the UTEM impinge on the Ag/Si_3_N_4_ film and interact with the modulated femtosecond light-pulse field at the Ag surface via stimulated inverse transition radiation. The inelastically scattered electrons are then imaged in space, time, and energy by means of electron energy-loss spectroscopy (EELS) performed in our EF-TEM setup. (b) Schematic picture of the optical modulation of a free electron wave by a shaped light wave with a Hermite-Gaussian transverse profile. (c) Sequence of measured EELS spectra (color map) plotted as a function of the delay time between the electron and light pulses. Sidebands at energies  (in our case, *ℏω* = 1.57 eV) relative to the zero-loss peak (ZLP) are visible, where  is the net number of exchanged photons. White solid line: selected EELS spectrum measured at *t* = 0, corresponding to the temporal and spatial coincidence between electron and light pulses.

In essence, we overcome the problem of designing and fabricating complicated electron-optic elements by instead shaping light beams, which has been proven a much easier task to perform. At the same time, the method allows an unprecedented access to fast temporal modulation. Indeed, a critical advantage of our approach with respect to existing technologies lies in the capability to achieve ultrafast switching of the electron wave profile and an extremely flexible electron manipulation. The fast, tailored, and versatile control achievable with such ultrafast light fields would allow us to simultaneously engineer the spatial, temporal, spectral, and momentum distributions of an electron in a coherent manner, providing new approaches for the investigation of ultrafast excitations in materials.^53−61^

As shown in Figure 1a,b, a suitable platform for generating the required light field configuration is provided by a tilted light-opaque, electron-transparent thin film in which an externally controlled optical pattern is projected from a SLM placed in the conjugate plane with respect to the film surface. In our design, we adopt a 30 nm thick Ag layer deposited on a 20 nm thick Si_3_N_4_ membrane. Femtosecond electron pulses impinge on the Ag/Si_3_N_4_ plate and interact with the semi-infinite light field created at the Ag surface via stimulated inverse transition radiation.^26^ The inelastically scattered electrons are then imaged in space and time by electron energy-loss spectroscopy (EELS; see the Methods section for further experimental details).

In Figure 1c, we show EELS spectra recorded as a function of the delay time between electron and light pulses. Following the interaction, the zero-loss peak (ZLP) at an electron energy *E*_0_ = 200 keV is redistributed among sidebands at multiples of the incident photon energy , corresponding to energy losses and gains by the electrons of  photon quanta (in our case *ℏω* = 1.57 eV). The electron–light interaction is captured by a single complex coupling coefficient^26,62^
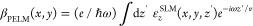
1where *v* is the electron speed and *e* is the elementary charge. The parameter β_PELM_ depends on the component of the light electric field ε_*z*_^SLM^ along the propagation direction *z* and on its distribution over the transverse (in-plane) coordinates (*x*, *y*). We remark that the transverse dependence is key for our photonic electron modulator.

Following the interaction with the optical field, the initial electron wave function ψ_0_ gains inelastic components labeled by the net number of photon exchanges  according to
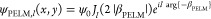
2where  is the ^th^-order Bessel function of the first kind, and we only show the in-plane wave function dependence.

In our approach, we use the SLM to imprint a transverse amplitude and phase pattern on the optical field, which is then directly transferred to β_PELM_(*x*, *y*) and, thus, to the amplitude and phase of the inelastic electron wave components (*x*, *y*). This is demonstrated here for the cases of Hermite-Gaussian and Laguerre-Gaussian modulation.

In Figure 2a–c, we show the phase patterns implemented on the SLM. When using a homogeneous phase distribution, the SLM behaves as a regular homogeneous mirror, and the Gaussian light pulses are thus focused unperturbed on the Ag/Si_3_N_4_ thin film (see Figure 2d). Instead, the introduction of a well-defined π-phase shift between two halves of the SLM, separated by a boundary along either the horizontal direction (Figure 2b) or the vertical direction (Figure 2c), induces the formation of Hermite-Gaussian (HG) optical beams in the conjugate plane with HG_10_ (Figure 2e) or HG_01_ (Figure 2f) symmetry, respectively.

**Figure 2:**
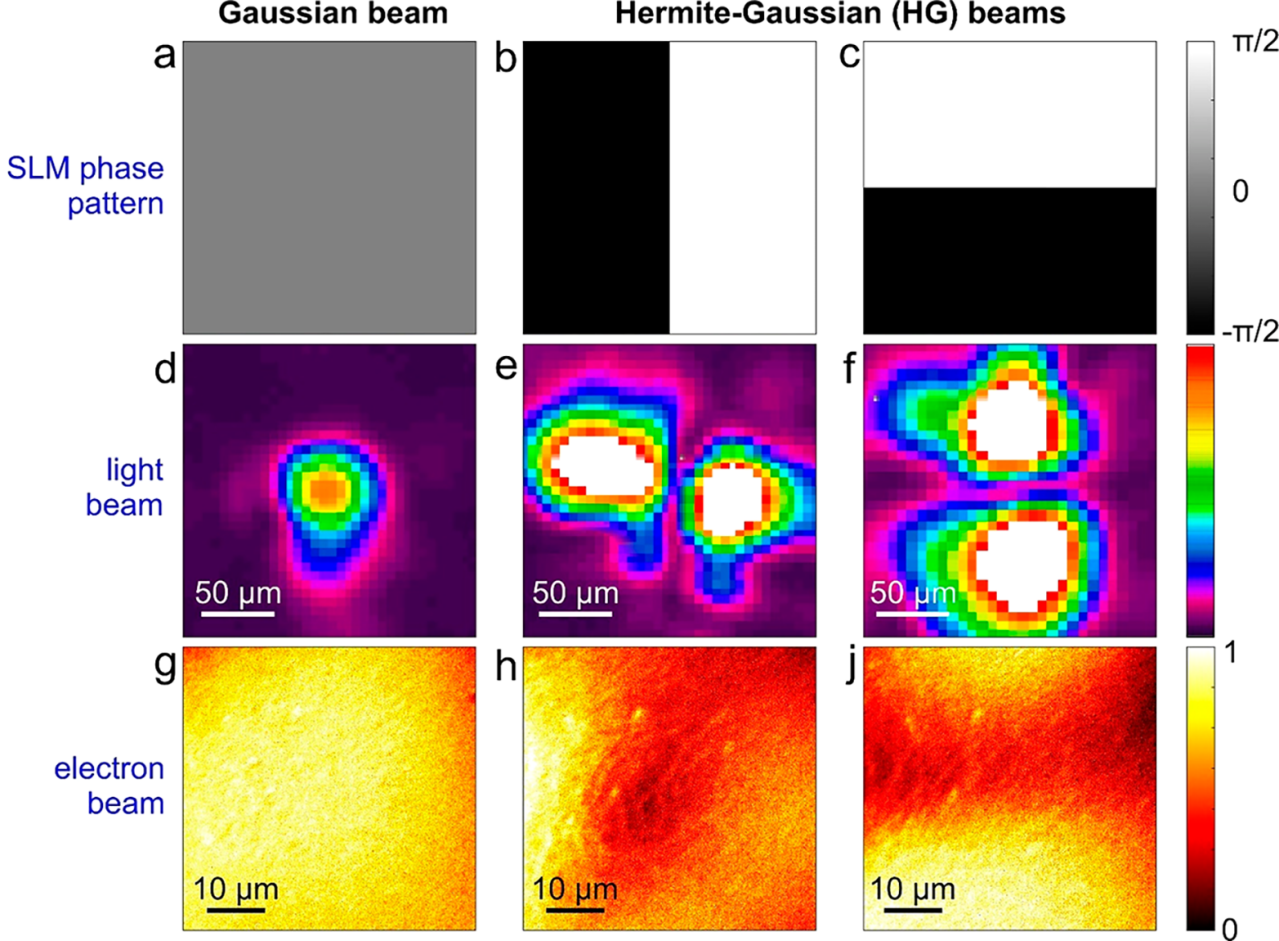
Experimental demonstration of transverse optical modulation of free electrons via arbitrarily shaped ultrafast light fields. (a–c) Phase patterns implemented on the SLM used to modulate the light field: (a) homogeneous phase distribution, (b) π-phase shift along the horizontal direction, and (c) π-phase shift along the vertical direction. (d–f) Light transverse profiles measured for the corresponding SLM phase patterns in (a)–(c): a Gaussian profile in (d), a two-lobed horizontal Hermite-Gaussian profile (HG_10_) in (e), and a two-lobed vertical Hermite-Gaussian profile (HG_01_) in (f). (g–j) Inelastically scattered electron spatial maps measured under optical illumination with (g) Gaussian and (h–j) Hermite-Gaussian beams, obtained by the SLM modulation of the ultrafast light pulses shown in (a)–(c). All images are taken when the electron and light wavepackets have maximum temporal overlap.

In Figure 2g–j, we show the inelastically scattered electron spatial maps, see Methods for details, measured under optical illumination with Gaussian and Hermite-Gaussian beams obtained by SLM modulation of the ultrafast light pulses. These images are taken when the electron and the light wavepackets are in temporal coincidence. Here, we immediately notice that the characteristic Hermite-Gaussian distributions are directly imprinted on the transverse profile of the electron pulse, which changes from a HG_10_ (Figure 2h) to a HG_01_ (Figure 2j) symmetry according to the in-plane pattern on the optical field.

As described in ref (24), the coupling coefficient β_PELM_ for the adopted experimental geometry of a semi-infinite light field reflected from a 30 nm thick Ag layer (treated as a perfect-conductor mirror) is given by
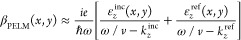
3where ε_*z*_^inc/ref^ and *k*_*z*_^inc/ref^ are the projections of the incident/reflected SLM light electric field and wave vector, respectively, on the *z* direction defined by the electron beam, which coincide with the plate surface normal in the present instance. These quantities depend on the tilting geometry of the layer with respect to the electron and light directions. Their detailed expressions for the geometrical parameters used in the experiments are offered in the Supporting Information, Section 1. In Figure 3d–f, we plot the calculated incident transverse profiles of the light pulse used for the experiments performed in this work, where either a Gaussian or a Hermite-Gaussian beam is made to interact with the electron pulse. Upon propagation of the electron wavepacket through such a field configuration, the inelastically scattered electron spatial maps can be obtained as
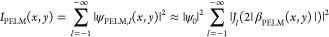
4where the summation runs over the energy gain components of the electron wave (i.e.,  < 0). The calculated maps are shown in Figure 3g–j and correctly reproduce the two-lobed probability distributions observed in experiment, as induced by the SLM phase modulation of the optical field (see also Supporting Information, Section 2 and Figure S2). It is worth mentioning that the tilt of the electron lobes is a result of the off-normal light incidence on the Ag mirror.

**Figure 3:**
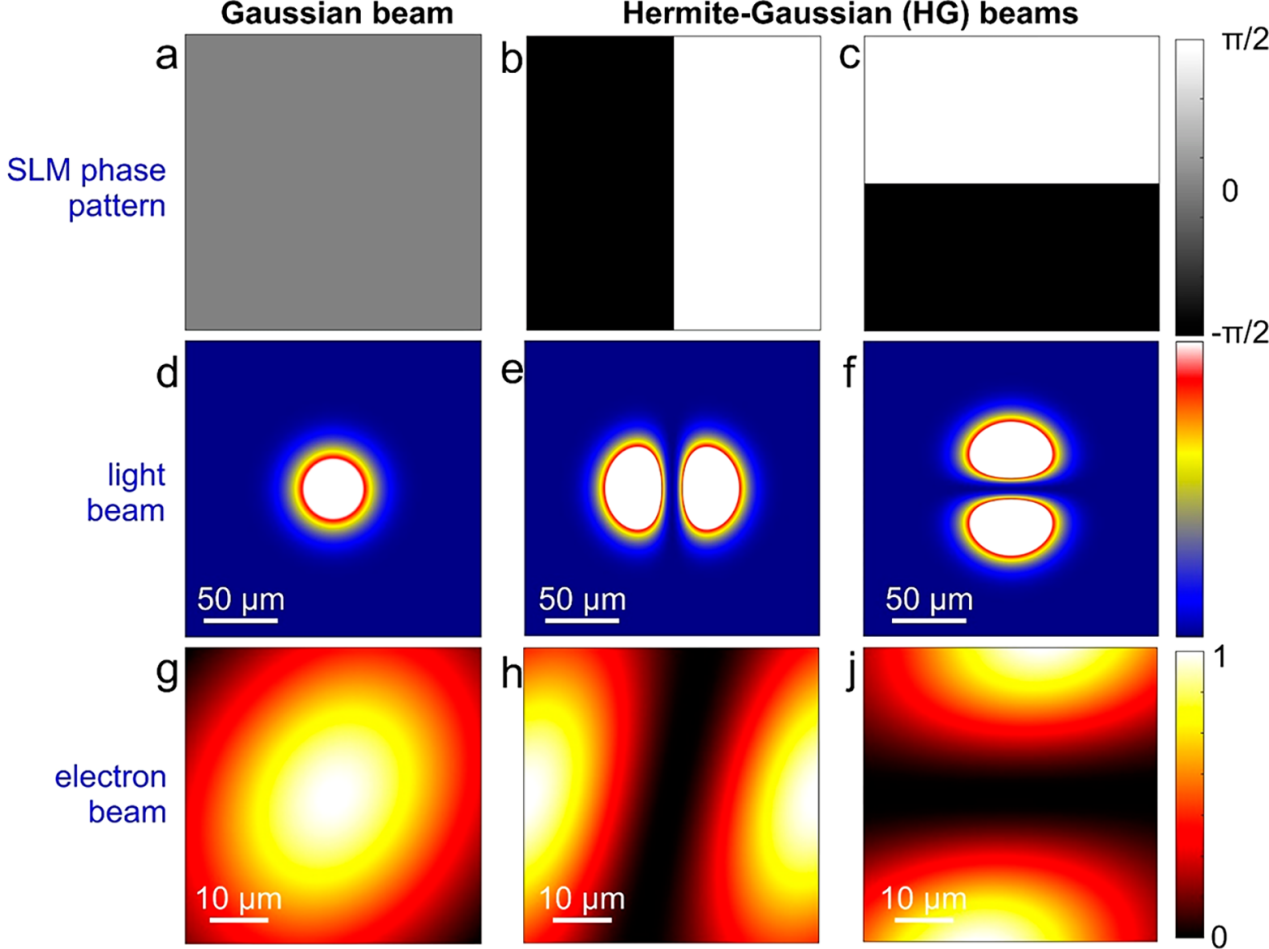
Theoretical calculations of transverse optical modulation of free electrons via arbitrarily shaped ultrafast light fields. We present simulations corresponding to the same plots and labels as in Figure 2. The asymmetry in the electron beam profiles in (g)–(j) is due to the tilt angle of the incident light direction relative to the electron beam

The approach demonstrated here is, of course, significantly more general than just the Hermite-Gaussian modulation shown above. As an example, we demonstrate in Figure 4 the vortex shaping of the electron beam starting from an optical Laguerre-Gaussian (LG) beam with azimuthal order equal to 1. In Figure 4a, we show the vortex phase pattern implemented on the SLM, whereas Figure 4b shows the optical beam profile at the conjugate plane where the Ag thin film resides. Finally, Figure 4c shows the corresponding energy-filtered electron image in which we observe the optical vortex pattern directly imprinted on the electron transverse profile.

**Figure 4:**
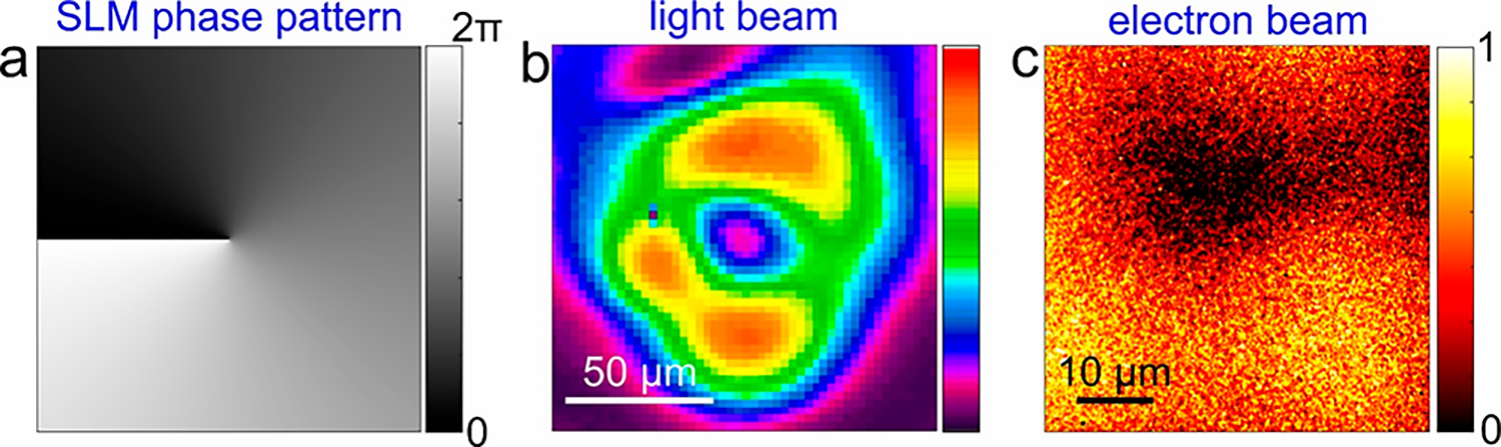
Ultrafast vortex modulation of free electron pulses via an optical Laguerre-Gaussian (LG) beam. (a) Vortex phase pattern implemented on the SLM with azimuthal order equal to 1. (b) Optical beam profile at the conjugate plane where the Ag thin film resides showing the optical LG mode. (c) Energy-filtered electron image where we observe the optical vortex pattern directly imprinted on the electron transverse profile.

Having demonstrated the ability to imprint a Hermite-Gaussian modulation on femtosecond electron beams, we discuss its possible application in the dynamical investigation of materials. Because HG beams are the electron equivalent of linearly polarized light, they are particularly sensitive to the mode symmetry of localized electromagnetic fields.^63,64^ For instance, ultrafast HG beams with a well-defined transverse phase coherence and azimuthal order could act as selective probes of the dynamical behavior of specific plasmonic resonances characterized by the corresponding charge multipolarity.^63,64^ These types of probes hold an enormous potential for the investigation of the spatiotemporal field evolution in photonic cavities and metamaterials, where multiple degenerate modes of different symmetry are usually excited at unison.

Additionally, shaped electron beams can also be used to enhance the contrast in TEM images from weak scatterers.^65−69^ This can lead either to a reduction of the total electron dose needed to form an image–thus reducing the radiation damage–or to an improvement (with the same electron dose) of the signal-to-noise-ratio in such an image. The latter aspect would be beneficial for the investigation of the real-time evolution of beam-sensitive samples in their natural environment.

As an example of the application of Hermite-Gaussian modulation in TEM imaging, we theoretically demonstrate the possibility of using HG beams to enhance the topographic contrast of an ordered array of magnetic skyrmions in the limit of the weak-phase approximation. In terms of electron shaping schemes, the Hermite-Gaussian modulation of the electron wavepacket induced by the PELM would be equivalent to the application of a Hilbert-phase plate (HPP) with the corresponding symmetry. The HPP, which imposes a π-phase shift in half of the interaction region, is a well-known and well-characterized type of phase plate that is used in imaging of weak-phase objects.^65−68^ For illustration, we consider a hexagonal array of skyrmions (diameter of ∼50 nm) separated by a distance of 100 nm and perform simulations using the STEM-CELL software.^70^ TEM imaging of skyrmions is generally performed in Lorentz microscopy in Fresnel mode with a defocus of hundreds of microns or more.^71,72^ In Figure 5a,b (see further details in the Methods section), we present the simulated TEM images of the skyrmion lattice obtained at the focus condition (zero defocus) with Hermite-Gaussian electron beams by the application of two orthogonal HPPs having the corresponding symmetries, respectively. In Figure 5c, we show the quadratic average of the two HG images. The latter provides a highly resolved image of the skyrmion lattice with a strong increase of the local contrast with respect to conventional LTEM images, where lateral resolution is limited by the large defocus. Such a method, which takes advantage of the unique possibility of PELM to rapidly alternate between the two symmetries of the HG beam, could be used as starting point for quantitatively reconstructing the phase of the transmitted electron-beam wave function following the interaction with the sample.

**Figure 5:**
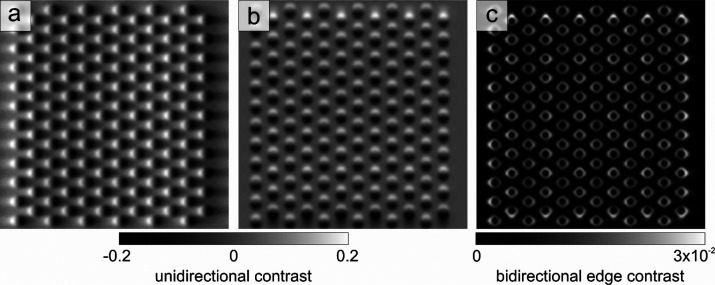
Theoretical calculations of topographical contrast enhancement of a weak-phase object using Hermite-Gaussian electron beams. (a, b) Simulated TEM images of an ordered array of Skyrmions (diameter of ∼50 nm, separation of 100 nm) obtained at the focus condition (zero defocus) with shaped electrons having (a) HG_10_ and (b) HG_01_ symmetries. The Hermite-Gaussian modulation of the electron wavepacket induced by the PELM is equivalent to the application of a Hilbert phase plate with the corresponding symmetry. (c) Quadratic average of the two images reported in panels (a) and (b), showing an enhanced local edge contrast of the array of Skyrmions. The field of view in all panels is 1.13 × 1.13 μm^2^.

Our results represent the first step toward the realization of an all-optical rapidly programmable phase mask for electrons. The inelastic interaction between an electron pulse and an arbitrarily shaped ultrafast light field controlled by an external spatial light modulator will allow us to achieve ultimate amplitude and phase control of the electron wavepacket in space and time. Besides the fundamental aspects, this will have a strong technological impact in enabling new imaging methods in electron microscopy with enhanced performances–especially in terms of sensitivity and resolution, which are hardly attainable based on conventional static or slowly varying schemes. We thus anticipate our approach to be a step forward in the ability to radically change how matter is investigated in electron microscopy.

As an appealing possibility, optically induced transverse amplitude modulation of electron wavepackets would be ideal for the implementation of an electron single-pixel imaging (ESPI) scheme in space and time,^61^ where we would use laterally structured electron pulses to illuminate the object of interest while synchronously measuring the total intensity of the inelastically scattered electrons. In a TEM, spatial and temporal SPI has not yet been implemented, mainly due to the lack of fast and versatile electron modulators capable of generating the required rapidly changing electron patterns. Therefore, our results are extremely promising in terms of the experimental realization of Electron-SPI, which will enable microscopic investigations with lower noise, faster response time, and lower radiation dose with respect to conventional imaging approaches.

An additional intriguing opportunity opened by the PELM design is the generation of ultrashort vortex electron pulses with a designated azimuthal order via interaction with vortex light pulses created by the SLM pattern. Such a configuration will allow us to implement time-resolved electron chiral dichroism, enabling ultrafast chiral sensitivity at the ultimate temporal and spatial resolutions. In comparison with conventional methods, the key aspect of our approach would be the possibility to temporally lock the SLM with the laser and the electron detector, providing fast vorticity switching of the electron pulse and homodyne extraction of the dichroic signal. This approach will thus provide a tool able to access the out-of-equilibrium behavior of chiral excitations in quantum materials–one of the current challenges in condensed-matter physics–such as chiral phonons in 2D van der Waals layers with broken space-inversion symmetry^73^ or chiral plasmons in Dirac systems.^74^

Besides the many advantages of our electron modulation approach, it is worth presenting a critical view of the issues that one has to face when using this method. First, the presence of the Ag/Si_3_N_4_ film has several adverse effects: (i) the nonideal light reflectivity from the silver layer creates a small loss of intensity (just a few %) for the incoming light beam; (ii) the film limits the maximum fluence that can be adopted for the incoming light beam to a value of the order of tens of mJ/cm^2^; (iii) the crossing of a material induces electron incoherent scattering and loss of electron beam intensity (by around several 10%). Also, a reduced intensity in the modulation pattern stems from energy filtering. In this respect, although the energy filter has to efficiently separate a given sideband with good efficiency to reduce noise, we note that a relatively modest reduction should be sufficient, as we estimate that ∼ 34% of the electron signal can be placed in each of the first (gain or loss) sidebands when properly tuning the laser excitation parameters (wavelength, fluence, and temporal duration). This is the case, for instance, of precise phase modulation where only a specific sideband has to be selected and it is not possible to simultaneously collect different sidebands at once. Indeed, when we are interested in intensity modulation, those two (gain or loss) first sidebands deliver the same pattern, and thus, 68% of the electrons would be contributing when properly suppressing the ZLP, making the loss of intensity not too severe.

Although our method requires energy filtering after the electron–light interaction at the PELM plane, this does not involve extremely high-energy resolution. In principle, a band-pass filter with a ∼1 eV range window (centered on the gain side) or even a band-stop filter with a ∼1 eV range window (centered on the ZLP) would be sufficient. Possible technological solutions in this direction could rely on, for instance, combined electrostatic-magnetostatic devices that can be directly used in the column before sample interaction. As a potential improvement, light patterns could also be engineered to eventually remove the need for energy filtering, or at least only require slight suppression of the ZLP. This is in fact possible when properly tuning the light field intensity and localization for interacting with the electron beam and modulating its energy distribution,^24,26^ resulting in a substantial suppression of the ZLP, while almost all intensity can be contained in the first gain and loss sidebands. In this situation, even in the absence of energy filtering, it would be possible to efficiently reconstruct the pattern with high contrast. Eventually, patterns could be designed taking into account all sidebands by modulating the SLM, so that a sufficiently high number of basis functions is constructed and projected on the specimen; this type of approach could be optimized, for example, using a deep-learning-based methods.

Finally, we briefly describe the application of our PINEM-based approach to transverse modulation of continuous electron beams. At the moment, this is not technologically straightforward. Nevertheless, several recent papers^75−78^ have shown different implementation of continuous wave (CW) PINEM, although their focus was on either performing electron energy−gain spectroscopy (EEGS) or modifying the longitudinal phase of the electron wave function, rather than the lateral beam profile. One recent example of such an inelastic electron-light interaction consisted in using microring optical cavities.^50^ In such a scheme, light is delivered through an optical fiber to the cavity and the electron beam then interacts with the evanescent optical field from the cavity. This configuration could potentially be used also for transverse electron shaping, provided that suitable cavity excitation is used and the SLM pattern is properly modified to compensate for the distortions introduced by the fiber propagation. Free-space CW modulation via a ponderomotive phase^42^ could be another option, although it requires a substantial development of light modulation at the large light intensities used, thus posing another set of challenges.

In conclusion, we have experimentally demonstrated the first steps toward the synthesis of transverse electron modulation with arbitrary profiles by utilizing ultrafast shaped optical fields and their inelastic interaction with the electron. When such a modulation is created before interacting with the sample, we expect this method to enable new imaging capabilities, improve our ability to visualize the dynamic behavior of nanoscale materials, and disentangle the interplay of their multiple degrees of freedom.

## Methods

### Sample Preparation

The thin plates used in the experiments are made of a 30 nm thick silver layer deposited via sputtering at a rate of 5.8 Å/s on a 20 nm thick Si_3_N_4_ membrane placed on a silicon support. The plate is mounted on a double-tilt TEM sample holder to ensure full rotation around the transverse *x* and *y* axes (see Supporting Information, Figure S1). In particular, the plate rotates with an angle ϑ around the *y* axis and an angle α around the *x* axis.

### UTEM Experiment

The experiments are performed in an ultrafast transmission electron microscope, whose technical details are described in ref (52). Briefly, we illuminate the thin Ag/Si_3_N_4_ film described above using 600 fs infrared pulses (*ℏω* = 1.57 eV photon energy) at a repetition rate of 1 MHz delivered by a KMLabs Wyvern X Ti:sapphire amplified laser system. The light propagates within the *y*–*z* plane and forms an angle δ ∼ 4.5° with the *z* axis, as shown in Supporting Information, Figure S1. For the experiments presented in the main text, we use a peak light-field amplitude ∼ 10^7^ V/m. The delay between electron and photon pulses is varied via a computer-controlled delay line. Simultaneously, a small portion of the infrared laser output is frequency-tripled and directed toward the LaB_6_ cathode of a JEOL JEM-2100 transmission electron microscope from which femtosecond single-electron pulses are generated via photoemission. The electron pulses are accelerated to an energy *E*_0_ = 200 keV along the *z* axis, and then focused onto the thin Ag/Si_3_N_4_ plate.

We use a light pulse duration of 600 fs (fwhm). For electrons, we estimate the pulse duration by measuring the electron–photon cross-correlation, as obtained by monitoring the EELS spectra as a function of the delay time between electrons and the infrared light (see Figure 1c). In the low-excitation regime, the measured temporal width of the th sideband is roughly , where *τ*_*e*_ and *τ*_*L*_ are the electron and optical pulse durations, respectively.^79^ For infrared pulses with *τ*_*L*_ = 600 fs, we derive an electron pulse duration *τ*_*e*_ = 670 fs fwhm.

Inelastically scattered electrons are then recorded in space and time with a Gatan imaging filter (GIF) spectrometer coupled to a K2 direct detector camera for energy-filtered real-space imaging and spectroscopy (∼1.2 eV energy resolution). The real-space images presented in this work are acquired in energy-filtered mode by selecting a ∼15 eV window centered on the energy gain side, leaving out the zero-loss peak (ZLP). This procedure directly provides the spatial distribution of the inelastically scattered electrons interacting with the optical fields at the silver surface of the thin film.

It is worth mentioning that the size of the field of view in the current experiment is dictated by the low numerical aperture of our current optical setup (although this is the state-of-the-art in ultrafast TEM). Future developments of the optical setup with higher numerical apertures, based for instance on plasmonic metalenses and miniaturized parabolic lenses placed directly into the TEM, are under consideration for practical implementation of the method.

### Spatial Light Modulation

As shown in Figure 1a, before reaching the Ag thin film inside the microscope, the infrared light beam is directed toward the surface of a SLM working in reflection mode. The SLM is a PLUTO 2.1 from HOLOEYE Photonics AG, suitably designed to modulate light close to 800 nm wavelength and featuring a high reflectivity and endurance to high light power. The SLM display, which features a resolution of 1920 × 1080 pixels with 8 μm pixel size, is addressed with phase functions via standard graphics cards as an extended monitor device. To ensure optimal light modulation efficiency, we introduce a polarizer to set the horizontal polarization state of the infrared light beam before reaching the SLM, and illuminate the SLM surface at an angle of ∼7° with respect to its normal. Following SLM modulation, the light field is then focused on the Ag/Si_3_N_4_ film by means of a plano-convex lens with a focal length of ∼25 cm. Such a configuration makes the silver surface the conjugate plane of the SLM surface. The spatial maps of the light transverse distribution are then measured via an optical beam profiler placed at a distance from the lens equivalent to the Ag surface and following a path that mimics the one traveled by the light beam within the microscope.

In our experiments we have chosen a nematic-based liquid crystal (LC) SLM. This is because, compared to ferroelectric-based LC SLMs or digital micromirror devices (DMDs), the nematic-based SLMs have higher resolution and can provide a better quality phase modulation. The switching time is related to the thickness of the LC layer performing the phase modulation (the thicker the cell, the slower the device, but the higher the dynamic range). For the SLM model used in the current work (phase modulation > 2π) and designed for the NIR wavelength range (between 800 and 1064 nm) typical switching times (rise and fall times) range between 50 and 180 ms.

### Electron Imaging Simulations

Simulations of electron microscopy images presented in Figure 4 are performed using the STEM-CELL software.^70^ The simulations are based on typical skyrmion visibility conditions as considered in the work from Pöllath et al.,^80^ and correspond to considering Bloch Skyrmions with a nearly-Gaussian phase profile and a maximum phase value of about 0.2 rad. Using the calibration curves in the reference above, this corresponds to a scenario with a magnetization of about 3 × 10^5^ A/m, a skyrmion radius of 50 nm, and a film thickness of 30 nm. The generic electro-optical configuration of the TEM is based on the Lorentz/Low-Mag mode, with the specimen placed in the standard sample plane and the electron modulation occurring in a conjugate plane before the sample position (i.e., the condenser aperture plane). For our PELM system, phase variations cannot occur on a scale shorter than the light wavelength, and thus, we assume a phase resolution in the modulation plane of the order of 1 μm. For UTEM imaging, we consider a lateral coherence length at the sample position of a few hundreds of nm’s (corresponding to a collimation angle of 1–10 μrad). The spherical aberration parameter is assumed to be Cs = 10 cm and the defocus value is set to zero because the best contrast with our PELM modulation is achieved under in-focus conditions.

## Supporting Information Available

Detailed description of the theoretical calculations of electron–light interaction and additional simulations of Hermite-Gaussian modulation of ultrafast electron pulses (PDF)
