# Supplementary material for: Ultrafast Transverse Modulation of Free Electrons by Interaction with Shaped Optical Fields
Source: ACS Photonics. 2022 Sep 27;9(10):3215–24. doi: 10.1021/acsphotonics.2c00850 (PMC9585634; doi:10.1021/acsphotonics.2c00850)
Supplement: Supplementary file 1 — ph2c00850_si_001.pdf [file ph2c00850_si_001.pdf]

Supporting Information

Ultrafast Transverse Modulation of Free Electrons by Interaction with Shaped Optical Fields

Ivan Madan[1], Veronica Leccese[1], Adam Mazur[2], Francesco Barantani[1,3], Thomas LaGrange[1], Alexey Sapozhnik[1], Phoebe M. Tengdin[1], Simone Gargiulo[1], Enzo Rotunno[4], Jean-Christophe Olaya[2], Ido Kaminer[5], Vincenzo Grillo[4], F. Javier García de Abajo[6,7], Fabrizio Carbone[1,*], and Giovanni Maria Vanacore[8,*]

1. Institute of Physics, École Polytechnique Fédérale de Lausanne, Lausanne, 1015, Switzerland
2. HOLOEYE Photonics AG, Volmerstrasse 1, 12489 Berlin, Germany
3. Department of Quantum Matter Physics, University of Geneva, 1211 Geneva, Switzerland
4. Centro S3, Istituto di Nanoscienze-CNR, 41125 Modena, Italy
5. Department of Electrical and Computer Engineering, Technion, Haifa 32000, Israel
6. ICFO-Institut de Ciencies Fotoniques, The Barcelona Institute of Science and Technology, 08860 Castelldefels (Barcelona), Spain
7. ICREA-Institució Catalana de Recerca i Estudis Avançats, Passeig Lluís Companys 23, 08010 Barcelona, Spain
8. Department of Materials Science, University of Milano-Bicocca, Via Cozzi 55, 20126 Milano, Italy

*To whom correspondence should be addressed: giovanni.vanacore@unimib.it, fabrizio.carbone@epfl.ch

SUPPLEMENTARY SECTION 1:
Theoretical calculation of electron-light interaction

The performance of the Ag/Si_3N_4 film used in the experiments is close to that of a perfect mirror (reflectivity larger than 99%). For our photon energy of 1.57 eV, the silver skin depth is ~ 11 nm, which is smaller than the silver layer thickness of ~ 30 nm. Under the approximation of a perfect mirror, the electron-light coupling β parameter is given by Eq. (3) in the main text. To compute such equation, we need to define the incident and reflected light electric fields and wave vectors for the tilting geometry adopted in the experiments.

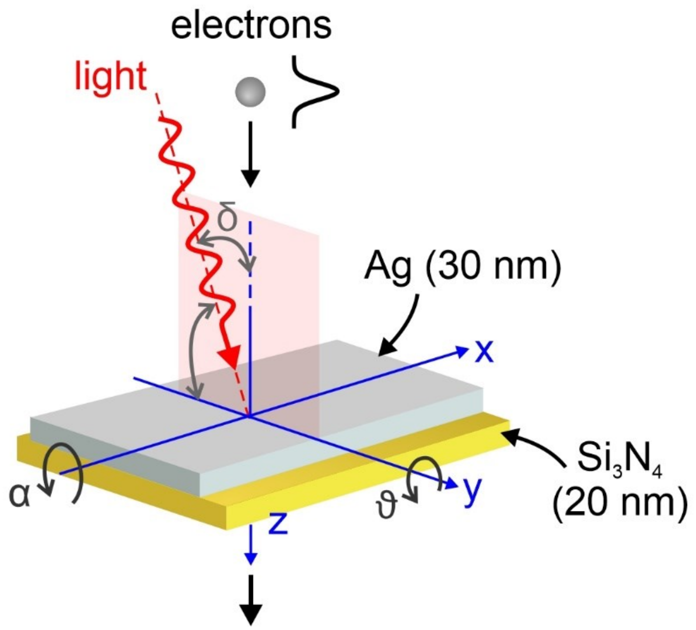

Figure S1. Schematic diagram of the experimental geometry. A Ag/Si_3N_4 plate is mounted on a double-tilt TEM sample holder to ensure full rotation around the transverse *x* and *y* axes. In particular, the plate rotates by an angle ϑ around the *y* axis and by an angle α around the x axis. The electron beam moves along the *z* axis, whereas the light beam propagates within the *y*–*z* plane and forms an angle δ ~ 4.5° with respect to the *z* axis.

Taking Fig. S1 as reference, we denote with $[x, y, z]$ the coordinates in the unrotated frame; $[x', y', z']$ in the frame after rotating by a tilt angle ϑ; and $[x'', y'', z'']$ in the frame after a subsequent rotation by an angle α. These two rotations are described by the relations:

$$x' = x\cos\vartheta + z\sin\vartheta; y' = y; z' = -x\sin\vartheta + z\cos\vartheta \quad \text{(S1)}$$

and

$$x'' = x';\ y'' = y'\cos\alpha - z'\sin\alpha; z'' = y'\sin\alpha + z'\cos\alpha. \quad \text{(S2)}$$

Combining Eq. (S1) and (S2), we find

$$x'' = x\cos\vartheta + z\sin\vartheta; y'' = y\cos\alpha - (z\cos\vartheta - x\sin\vartheta)\sin\alpha,$$
$$z'' = y\sin\alpha + (z\cos\vartheta - x\sin\vartheta)\cos\alpha. \quad \text{(S3)}$$

The incident light electric field and wave vector in the unrotated frame can be written as

$$\mathcal{E}^{inc}(x,y) = A\mathcal{E}_0(x,y)\begin{bmatrix}0\\ \cos\delta\\ \sin\delta\end{bmatrix},$$
$$k^{inc} = k_0\begin{bmatrix}0\\ \sin\delta\\ \cos\delta\end{bmatrix}. \quad \text{(S4)}$$

By applying the transformation defined by Eq. (S3), we obtain

$$\mathcal{E}^{inc''}(x'',y'') = A\mathcal{E}_0''(x'',y'')\begin{bmatrix}\sin\delta\sin\vartheta\\ \cos\delta\cos\alpha - \sin\delta\cos\vartheta\sin\alpha\\ \cos\delta\sin\alpha + \sin\delta\cos\vartheta\cos\alpha\end{bmatrix},$$
$$k^{inc''} = k_0\begin{bmatrix}\cos\delta\sin\vartheta\\ \sin\delta\cos\alpha - \cos\delta\cos\vartheta\sin\alpha\\ \sin\delta\sin\alpha + \cos\delta\cos\vartheta\cos\alpha\end{bmatrix}. \quad \text{(S5)}$$

Following the reflection from the (ideal) silver surface, the *x* and *y* components of the reflected quantities are the opposite of the *x* and *y* components of the incident quantities, while the *z* component remains the same. The reflected light electric field and wave vector are thus given by

$$\mathcal{E}^{ref''}(x'',y'') = A\mathcal{E}_0''(x'',y'')\begin{bmatrix}-\sin\delta\sin\vartheta\\ -\cos\delta\cos\alpha + \sin\delta\cos\vartheta\sin\alpha\\ \cos\delta\sin\alpha + \sin\delta\cos\vartheta\cos\alpha\end{bmatrix},$$
$$k^{ref''} = k_0\begin{bmatrix}-\cos\delta\sin\vartheta\\ -\sin\delta\cos\alpha + \cos\delta\cos\vartheta\sin\alpha\\ \sin\delta\sin\alpha + \cos\delta\cos\vartheta\cos\alpha\end{bmatrix}. \quad \text{(S6)}$$

Considering that the unit vector along the *z* axis, which we denote as u_z, is transformed from [0,0,1] in the unrotated frame to $u_z'' = [\sin\vartheta, -\cos\vartheta\sin\alpha,\ \cos\vartheta\cos\alpha]$ following α and ϑ rotations, we can calculate the projection of the incident/reflected electric field and wave vector in the rotated frame as

$$\mathcal{E}_z^{inc''} = \mathcal{E}^{inc''} \cdot u_z'' = A\mathcal{E}_0''\sin\delta,$$
$$k_z^{inc''} = k^{inc''} \cdot u_z'' = k_0\cos\delta, \quad \text{(S7)}$$

$$\mathcal{E}_z^{ref''} = \mathcal{E}^{ref''} \cdot u_z'' = A\mathcal{E}_0''[\sin\delta(\cos^2\vartheta\cos2\alpha - \sin^2\vartheta) + \cos\delta\cos\vartheta\sin2\alpha],$$
$$k_z^{ref''} = k^{ref''} \cdot u_z'' = k_0[\cos\delta(\cos^2\vartheta\cos2\alpha - \sin^2\vartheta) + \sin\delta\cos\vartheta\sin2\alpha]. \quad \text{(S8)}$$

Finally, the transverse light profile in the unrotated frame is given by either a Gaussian distribution or an Hermite-Gaussian distribution according to the settings used for the SLM:

$$\mathcal{E}_0^G(x,y) = \exp\left[-\frac{(x^2+y^2)}{2\sigma_L^2}\right],$$
$$\mathcal{E}_0^{HG10}(x,y) = \exp\left[-\frac{(x^2+y^2)}{2\sigma_L^2}\right]\frac{2x}{\sigma_L}, \quad \text{(S9)}$$
$$\mathcal{E}_0^{HG01}(x,y) = \exp\left[-\frac{(x^2+y^2)}{2\sigma_L^2}\right]\frac{2y}{\sigma_L},$$

where $2\sqrt{2ln2}\sigma_L$ defines the full width at half maximum (FWHM) of the laser beam. By applying the transformation defined by Eq. (S3) with *z* = 0, we can retrieve the transverse light profiles in the rotated frame:

$$\mathcal{E}_0^{G''}(x'',y'') = \exp\left[-\frac{\left(\Gamma_1 x''^2 + \Gamma_2 y''^2 - 2\Gamma_3 x''y''\right)}{2\sigma_L^2}\right],$$
$$\mathcal{E}_0^{HG10''}(x'',y'') = \exp\left[-\frac{\left(\Gamma_1 x''^2 + \Gamma_2 y''^2 - 2\Gamma_3 x''y''\right)}{2\sigma_L^2}\right]\frac{2x''}{\cos\vartheta\sigma_L}, \quad \text{(S10)}$$
$$\mathcal{E}_0^{HG01''}(x'',y'') = \exp\left[-\frac{\left(\Gamma_1 x''^2 + \Gamma_2 y''^2 - 2\Gamma_3 x''y''\right)}{2\sigma_L^2}\right]\frac{2}{\sigma_L}\left(\frac{y''}{\cos\alpha} - \tan\vartheta\tan\alpha x''\right),$$

where

$$\Gamma_1 = \frac{1}{\cos^2\vartheta} + \tan^2\vartheta\,\tan^2\alpha,$$
$$\Gamma_2 = \frac{1}{\cos^2\alpha}, \quad \text{(S11)}$$
$$\Gamma_3 = \frac{\tan\vartheta\tan\alpha}{\cos\alpha}.$$

Calculations of the inelastically-scattered electron spatial maps are then implemented by plugging the expressions found in Eqs. (S7), (S8), (S10), and (S11) within Eqs. (3) and (4) in the main text, using the following geometrical and experimental parameters: $\delta = 4.5°$, $\alpha = 12.7°$, $\vartheta = 35°$, $\sigma_L = 23.3$ µm, *v* = 0.7c, and $A = 10^7$ V/m.

SUPPLEMENTARY SECTION 2:
Calculations of Hermite-Gaussian modulation of ultrafast electron pulses

In this section, we present additional calculations of the transverse electron distribution using Eq. (2) in the main text for light-mediated modulation with a Hermite-Gaussian laser beam. In particular, we consider the electron distribution associated with each photon order, or equivalently, with each PINEM peak in the spectrum (for simplicity we consider just the first five orders).

As it can be seen from Fig. S2, the two-lobe intensity pattern is maintained for each photon order (going from $\ell = 1$ on the left to $\ell = 5$ on the right), with the main variation being that the total intensity decreases for increasing orders – as expected. Also, the phase shift between the two lobes is maintained and scales with integer multiples of π according to the photon order: it is π for $\ell = 1$, 2π for $\ell = 2$, 3π for $\ell = 3$, 4π for $\ell = 4$, 5π for $\ell = 5$, and so on. Actually, we believe that such phase-shift increase is an advantage and can play a key role in actual applications of shaped electron beams for contrast enhancement in weak scatterers.

These calculations imply that the two-lobe structure of a Hermite-Gaussian beam is transferred also to the electron beam and it is maintained even when summing over multiple photon orders.

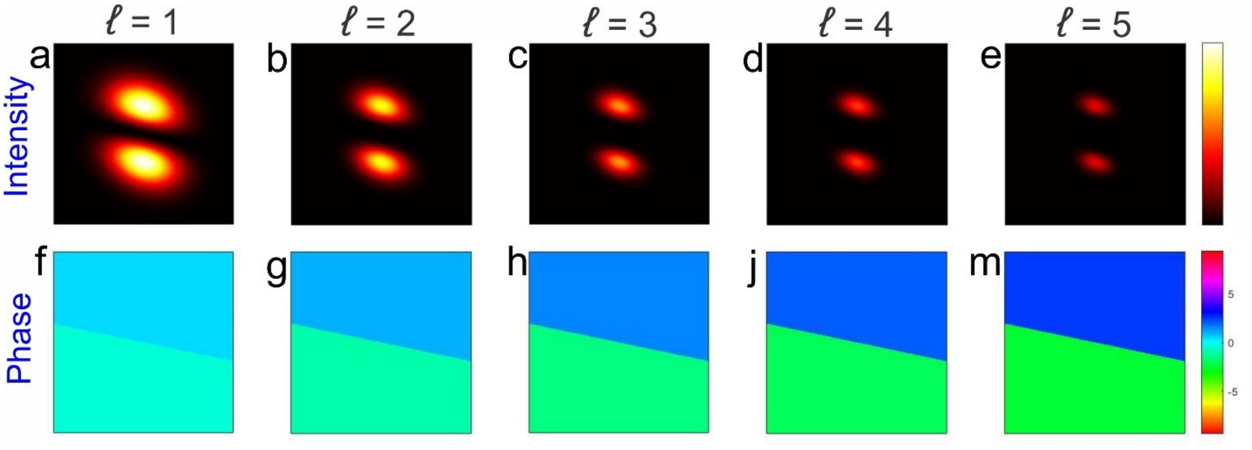

Figure S2. Calculated transverse electron distribution for light-mediated modulation with a Hermite-Gaussian laser beam as a function of the absorbed photon order ℓ. Panels (a)-(e) show the intensity distribution, whereas panels (f)-(m) represent the phase pattern.
